# Population structure and genome characterization of local pig breeds in Russia, Belorussia, Kazakhstan and Ukraine

**DOI:** 10.1186/s12711-016-0196-y

**Published:** 2016-03-01

**Authors:** Aleksei Traspov, Wenjiang Deng, Olga Kostyunina, Jiuxiu Ji, Kirill Shatokhin, Sergey Lugovoy, Natalia Zinovieva, Bin Yang, Lusheng Huang

**Affiliations:** L.K. Ernst Institute for Animal Husbandry, Dubrovitzy 60, Podolsk district, Moscow region Russia 142132; National Key Laboratory for Swine Genetics, Breeding and Production Technology, Jiangxi Agricultural University, Nanchang, 330045 People’s Republic of China; Siberian Research Institute for Animal Husbandry, Russian Agricultural Academy, Novosibirsk, Russia 63050; Mykolayiv National Agrarian University, 9, Paryzka Komuna Str., Mykolayiv, 54020 Ukraine

## Abstract

**Background:**

It is generally accepted that domestication of pigs took place in multiple locations across Eurasia; the breeds that originated in Europe and Asia have been well studied. However, the genetic structure of pig breeds from Russia, Belorussia, Kazakhstan and Ukraine, which represent large geographical areas and diverse climatic zones in Eurasia, remains largely unknown.

**Results:**

This study provides the first genomic survey of 170 pigs representing 13 breeds from Russia, Belorussia, Kazakhstan and Ukraine; 288 pigs from six Chinese and seven European breeds were also included for comparison. Our findings show that the 13 novel breeds tested derived mainly from European pigs through the complex admixture of Large White, Landrace, Duroc, Hampshire and other breeds, and that they display no geographic structure based on genetic distance. We also found a considerable Asian contribution to the miniature Siberian pigs (Minisib breed) from Russia. Apart from the Minisib, Urzhum, Ukrainian Spotted Steppe and Ukrainian White Steppe breeds, which may have undergone intensive inbreeding, the breeds included in this study showed relatively high genetic diversity and low levels of homozygosity compared to the Chinese indigenous pig breeds.

**Conclusions:**

This study provides the first genomic overview of the population structure and genetic diversity of 13 representative pig breeds from Russia, Belorussia, Kazakhstan and Ukraine; this information will be useful for the preservation and management of these breeds.

**Electronic supplementary material:**

The online version of this article (doi:10.1186/s12711-016-0196-y) contains supplementary material, which is available to authorized users.

## Background

The pig (*Sus scrofa*) is an important farm animal, as well as a relevant biomedical model for humans. The pig was independently domesticated in China and Europe approximately 9000 years ago [1–3] and has undergone subsequent environmental and artificial selection, which contributed to the formation of many breeds with different global characteristics. Population genetic parameters of pig breeds from Asia, Europe and America have been analyzed in detail using high-density single nucleotide polymorphisms (SNPs) [4–7], whereas the genetic diversity, population structure and evolution history of pig breeds from the large and climatically diverse Eurasian regions of Russia, Belorussia, Kazakhstan and Ukraine remain poorly studied.

Until the twentieth century, Russian pig production occurred on a relatively small scale, with an estimated national herd of approximately 20 million pigs. Most pigs were imported from either Great Britain or China and were raised by small families or landlords [8]. Pig breeding activity began in the late nineteenth century and mainly took place in west Russia, north Caucasus, the Baltic States, Belorussia and Ukraine. Many of the pig breeds that were formed and registered in the mid-twentieth century were generated by crossbreeding multiple foundation populations from various breeds, including Large White, Landrace, Middle White, Hampshire, Berkshire, Poland China, Tamworth, Mangalitsa, local wild boars and Asian pigs [9]. Twenty-two local breeds were recorded in the Soviet Union in 1980, including the Ukrainian White Steppe, Caucasian, Mirgorod, Urzhum, and Semirechensk breeds [9]. These breeds accounted for 29 million of the 73 million pigs in the Soviet Union in 1980 and played important roles in pork production and economic development at the local level.

Although pigs in Russia, Belorussia, Kazakhstan and Ukraine originated from imported breeds, after their introduction, they adapted to the local climate, poor quality feed and resident pathogens, which led to the development of breeds with unique characteristics that differed from those of the founding stocks. One good example is the Ukrainian White Steppe local breed, which was originally bred from Large White pigs as founders in 1928, has adapted well to the local environment and available feed and has resulted in a more robust breed with a rougher physique than the Large White breed [10]. The Semirechensk pig was created by crossing Kemerovo pigs with Large White and wild boars in Kazakhstan, and is highly adapted to extreme temperatures that range from 48 °C in summer and −50 °C in winter and to sharp change of temperatures within a day, e.g., from 5 °C at mid-night to 48 °C at mid-day in summer, which is typical of this region [11]. Red White Belted pigs have been developed since 1994 through the complex crossing of six breeds, including the Large White, Duroc, Mirgorod, Landrace, Pietrain, and Hampshire breeds [12]. The Red White Belted breed retains the white belt characteristic of the Hampshire breed and the red coat color of the Duroc breed [8]. While most of the breeds were developed for pork production, the miniature Siberian swine (Minisib) was selected for its small body size, which is well adapted to laboratory conditions. Minisib pigs have an average weight of about 50 kg at the age of 300 days [13]. Overall, pig breeds from Russia, Belorussia, Kazakhstan and Ukraine provide ideal material to study the genetic basis of environmental adaptation and phenotypic variation. However, over the last three decades, many of these breeds that are well adapted to the local climate and harsh feed, are resistant to diseases, and produce good quality meat, have been marginalized or replaced by international commercial breeds with higher productivity [9].

Genetic studies using high-density SNPs can provide insights into the genetic structure of these pig populations that can contribute to improve breeding and preservation programs for these local breeds [14, 15]. In this study, we analyzed 60K SNP genotypes of 13 representative pig breeds from western and eastern regions of Ukraine and Belorussia, north Kazakhstan, and western Siberia to explore their genetic diversity, population structure and evolutionary history.

## Methods

### Animals

**Fig. 1:**
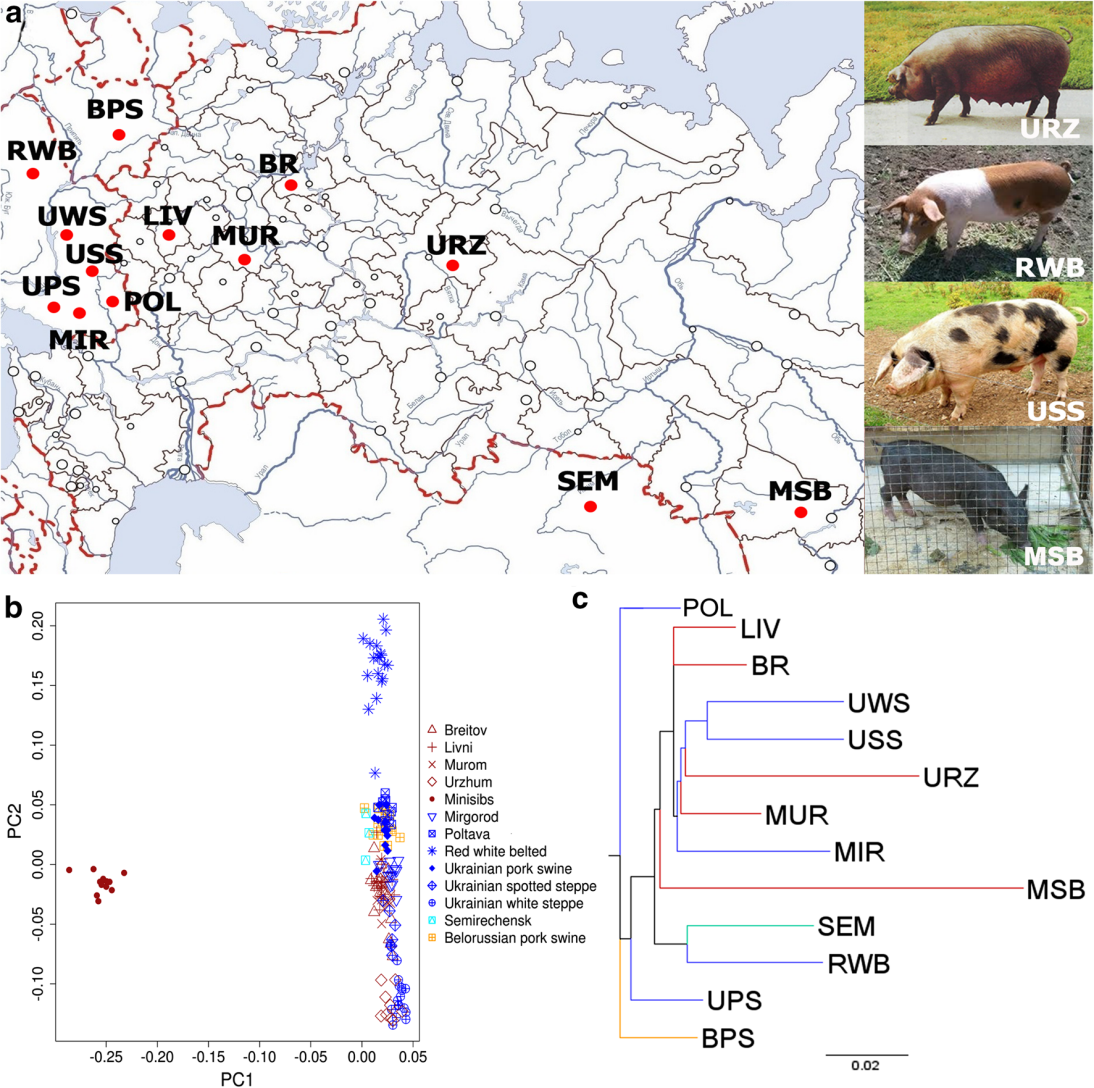
General information on the 13 breeds investigated. **a** Geographical distribution of native breeds from the Russian, Ukrainian, Belorussian and Kazakhstan populations. **b** Principal component analysis of 169 individuals from the 13 breeds under study. **c** Neighbor-joining tree of the 13 breeds based on *F*_ST_

A total of 170 pigs representing 13 breeds from Russia, Belorussia, Kazakhstan and Ukraine were sampled including five Russian breeds (Breitov, n = 18; Livni, n = 16; Minisib, n = 14; Murom, n = 12 and Urzhum, n = 9), one breed from Belorussia (Belorussian pork swine, n = 16), one breed from Kazakhstan (Semirechensk, n = 3), and six breeds from Ukraine (Mirgorod, n = 13; Poltava, n = 13; Red-White Belt, n = 19; Ukrainian pork swine, n = 12; Ukrainian Spotted Steppe, n = 7 and Ukrainian White Steppe, n = 18) (Table 1; Fig. 1a) and (see Additional file 1: Table S1). All samples were collected according to the guidelines for the care and use of experimental animals established by the Ministry of Agriculture of China. Unrelated samples were used for genotyping whenever possible to better represent the breeds under study. DNA was extracted from ear samples using a QIAGEN kit according to the manufacturer’s protocols and diluted to 20 ng/μL for genotyping [5]. DNA samples were genotyped for 61,565 SNPs using the Porcine SNP60 BeadChip (Illumina, San Diego, USA) [16].

**Table 1:** Genetic diversity of the 26 pig populations in this study

Breed	Code	Origin	Number^a^	ROH (Mb)	Ne	A_R_	H_O_
Pig breeds from Russia, Belorussia, Kazakhstan and Ukraine							
Belorussian pork swine	BPS	Belorussia	16	39	145.8	1.95	0.36
Breitov	BR	Russia, Yaroslavl region	18	60	148.7	1.95	0.35
Livni	LIV	Russia, Orel region	16	39	136.1	1.96	0.36
Murom	MUR	Russia, Vladimir region	12	30	86.2	1.93	0.36
Mirgorod	MIR	Ukraine, Poltava region	13	62	124.2	1.89	0.33
Poltava	POL	Ukraine, Poltava region	13	63	124.2	1.92	0.35
Red White Belted	RWB	Ukraine, Nikolaev region	19	68	142.8	1.96	0.35
Semirechensk	SEM	Kazakhstan, Southeast	3	21	–	1.75	0.38
Ukrainian pork swine	UPS	Ukraine, Askaina Nova	12	31	115.4	1.92	0.36
Ukrainian spotted steppe	USS	Ukraine, Askaina Nova	7	94	61.1	1.82	0.32
Ukrainian white-steppe	UWS	Ukraine, Askaina Nova	18	104	140.7	1.91	0.32
Urzhum	URZ	Russia, Kirov region	9	148	–	1.78	0.29
Minisib	MSB	Russia, Novosibirsk	14	181	99.5	1.81	0.3
Chinese breeds							
Erhualian	EHL	Wuxi, Jiangsu	32	63	164.9	1.72	0.19
Hetao large ear	HTDE	Wuyuan, Inner Mongolia	16	89	98.3	1.81	0.28
Luchuan	LUC	Luchuan, Guangxi	18	76	135.9	1.56	0.17
Laiwu	LWH	Laiwu, Shandong	18	189	118.4	1.77	0.23
Min	MIN	Lanxi, Heilongjiang	22	135	107	1.77	0.27
Tibetan (Tibet)	ZZT	Tibet	29	84	126.9	1.73	0.23
International commercial breeds							
Duroc	DRC	USA	35	157	207.2	1.88	0.27
Hampshire	HS	Britain	14	183	117.3	1.76	0.25
Landrace	LR	Denmark	35	108	207.5	1.97	0.31
Iberian	IB	Spain	16	200	122.4	1.81	0.19
Sicilian	SI	Italy	4	55	–	1.74	0.3
Bisaro	BI	Portugal	14	85	156.9	1.9	0.33
Large White	LW	Britain	35	75	214.4	1.99	0.33

*ROH* runs of homozygosity, *Ne* average effective population size, *A*_*R*_ allele richness, *H*_*o*_ observed heterozygosity

^a^Number of samples

### Data

The 60K SNP genotype data that were generated for the 170 pigs used in our study were combined with the genotype data from 288 pigs representing six breeds from China and seven international commercial or European breeds for comparison (Table 1), which resulted in a final dataset of 458 individuals. Quality control procedures were carried out using PLINK v1.9 [17]; briefly, individuals with genotype call rates lower than 0.9 (i.e. one Ukrainian pig individual) and SNPs with call rates lower than 0.9 or minor allele frequencies (MAF) lower than 0.05 were excluded. A total of 44,334 SNPs from 457 pigs representing 26 breeds were used for subsequent analyses.

### Genetic distances and population structure

PLINK v1.9 [17] was used to construct an identity-by-state (IBS) matrix, which quantifies the genetic similarity between individuals. Weir and Cockerham’s average *F*_ST_ [18] for SNPs across the genome was used to measure the genetic distance between a given pair of populations. Principal component analysis (PCA) was conducted using PLINK v1.9 based on the variance-standardized relationship matrix, which is the same as that computed by the GCTA software [17, 19]. To visualize the results, we created a scatterplot of the first and second principal components. Neighbor-joining trees were constructed using the “neighbor” program in PHYLIP v3.695 [20] and visualized using the FigTree v1.4.2 software [21]. Admixture analysis was conducted using the ADMIXTURE software v1.20 [22].

### Genetic diversity and effective population size

Allelic richness (A_R_) values were calculated using the ADZE v1.0 software [23]. Observed heterozygosity (H_o_) and runs of homozygosity (ROH) were computed with the PLINK v1.9 software [17], using 50-SNP sliding windows and allowing one heterozygote and five missing calls per window. The minimum length of a ROH segment was set to 500 kb. We calculated the sum of ROH per animal and the ROH of a population as the average percentage of the genome covered by ROH across all individuals of that population. The linkage disequilibrium (r^2^) between pairwise SNPs was calculated by the commands –r2 and –ld-window-r2 in PLINK v1.9 [17]. Within each breed, SNPs with a MAF higher than 0.05 and a missing data rate lower than 0.1 were used to calculate r^2^. Effective population sizes (Ne) were computed using the equation of Herrero-Medrano et al. [14] and Sved [24], i.e.: ${\text{Ne}}_{\text{T}} = \left( {1/4{\text{c}}} \right)*(1/{\text{r}}_{\text{c}}^{2} - 1)$, where Ne_T_ is effective population size at T generation ago, T is calculated by T = 1/2c [24], c is the genetic distance in Morgan, which was calculated by multiplying the physical distance (Mb) and recombination rate (Morgan/Mb) [25] between a pair of SNPs, and ${\text{r}}_{\text{c}}^{2}$ is the linkage disequilibrium between SNPs with c being the genetic distance. Thus, r^2^ between SNPs separated by large and small genetic distances reflect recent and ancient Ne, respectively [24].

## Results

### Genetic distances and population structures

Most of the pig breeds included in this study originated from west Russia, Belorussia and Ukraine, which were the main centers of agricultural production in the Soviet Union, since pig breeding in the eastern and Siberian regions of Russia was largely hampered by harsh climatic conditions. The first axis of the PCA plot of the 13 breeds (Fig. 1a) clearly separate the Minisib breed from the other breeds (Fig. 1b), which indicates that the genetic distance between the Minisib breed and the other 12 breeds is large. Among these 12 breeds, Red White Belted pigs were differentiated from the other breeds on the second axis (Fig. 1b). Both the PCA and the dendrogram of *F*_ST_ estimates show that the breeds did not cluster by geographical origin (Fig. 1b, c). For instance, the six Ukrainian breeds did not form a cluster although they are geographically close, whereas the Semirechensk and Red White Belted breeds are geographically distant but genetically similar.

**Fig. 2:**
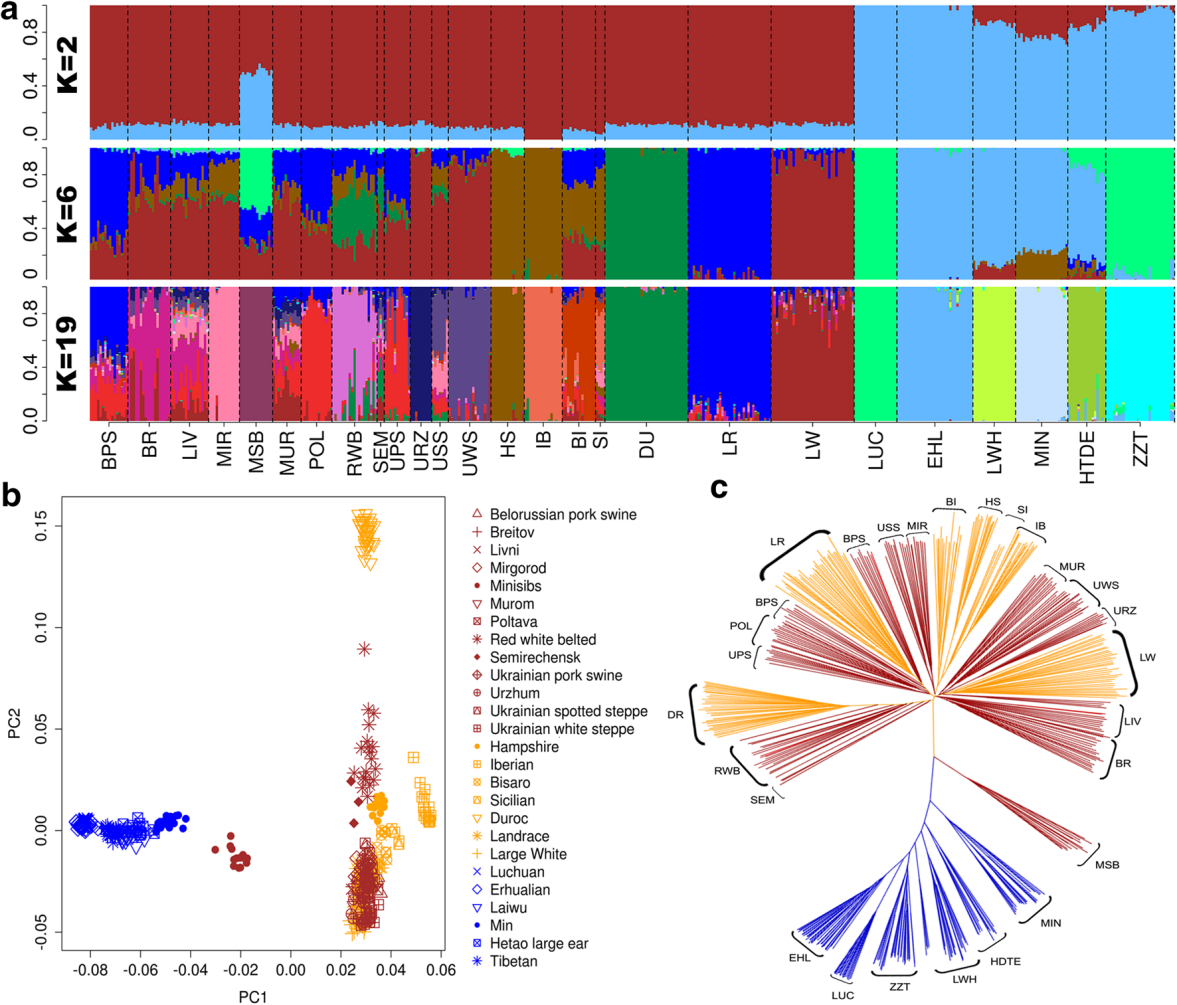
Analysis of the population structure of the 13 breeds. **a** *Bar plot* of ancestry compositions by ADMIXTURE with the assumed number of ancestries K = 2, 6, and 19. **b** Principal component analysis of 457 individuals from 26 breeds. **c** Neighbor-joining tree of 457 individuals from 26 breeds

The genetic structure of these 13 pig breeds was further investigated by comparing it with that of European and Chinese pig breeds (Table 1). As expected, admixture, PCA and neighbor-joining tree analyses clearly divided the European and Asian breeds into two clusters (Fig. 2). We observed widespread Asian ancestries in all 13 breeds under study (Fig. 2a, K = 2). Apart from the Minisib breed, which was positioned between the Asian and European groups (Fig. 2b) and could be traced back to Asia for about half of its ancestry, the other breeds clustered with the European pig breeds (Fig. 2b, c). This suggests that they are mainly of European origin, harboring different fractions of ancestry from the Large White, Landrace, Duroc and Hampshire breeds (Fig. 2a, K = 6). In spite of the presence of admixed ancestries from multiple international commercial breeds in all 13 breeds examined (Fig. 2a, K = 6), the neighbor-joining tree analysis showed that individuals from the same breed usually clustered together (Fig. 2c), which means that they have retained unique breed identities. Thus, between-breed genetic distances were always greater than within-breed genetics distances for these 13 breeds.

Among the 13 pig breeds investigated, the most striking one is the Minisib breed, which is characterized by a small body size (~50 kg at 300 days of age). The admixture analysis suggests that the Minisib breed contains ancestries from Large White, Landrace and Luchuan or Tibetan pigs from China (Fig. 2a, K = 6). This agrees with results of the PCA and the neighbor-joining tree analyses, which positioned the Minisib breed between the Chinese and European clusters (Fig. 2b, c). The Red White Belted and Semirechensk breeds were placed between the Duroc and Large White breeds on the second axis of the PCA plot (Fig. 2b) and were differentiated from the other ten breeds from western Russian, Belorussia and Ukraine, which all clustered with the Large White and Landrace breeds (Fig. 2b). This can be explained by the results of the admixture analysis, which show that the Red White Belted and Semirechensk breeds harbor considerable proportions of ancestry from the Duroc breed. The Duroc was bred in the United States and is genetically differentiated from other European breeds such as Large White and Landrace [26]. The Red White Belted pigs were also shown to harbor ancestry from the Hampshire breed (Fig. 2a, K = 6). Interestingly, this breed appears to have inherited both the red coat color from the Duroc breed and a white belt from the Hampshire breed (Fig. 1).

We ran the admixture analysis from K = 2 to 26 and found that K = 19 was the optimal value, with the smallest cross-validation errors (see Additional file 2: Figure S1) and (Fig. 2a). Among the 19 ancestries, 12 were from the group of breeds used for comparison, while seven were from the 13 breeds under investigation. Several breeds, including the Mirgorod, Minisib, Urzhum and Ukrainian White Steppe breeds, formed unique homologous ancestries (Fig. 2a, K = 19). The other breeds contained complex admixed ancestries from either the European commercial breeds or ancestries from the 13 breeds under study.

### Genetic diversity and demographic history

The allelic richness (A_R_) and observed heterozygosity (H_o_) were calculated to provide measures of the genetic diversity of the 13 populations (Table 1). We observed that the pig breeds from Russia, Belorussia, Kazakhstan and Ukraine generally showed higher levels of A_R_ and H_o_ than the Chinese and international commercial pig breeds. For instance, eight of the 13 breeds had average H_o_ values greater than 0.35, while all Chinese pigs had smaller H_o_ values, ranging from 0.17 for Luchuan pigs to 0.28 for Hetao pigs. The European pig breeds had H_o_ values ranging from 0.19 for Iberian pigs to 0.33 for Bisaro and Large White pigs.

**Fig. 3:**
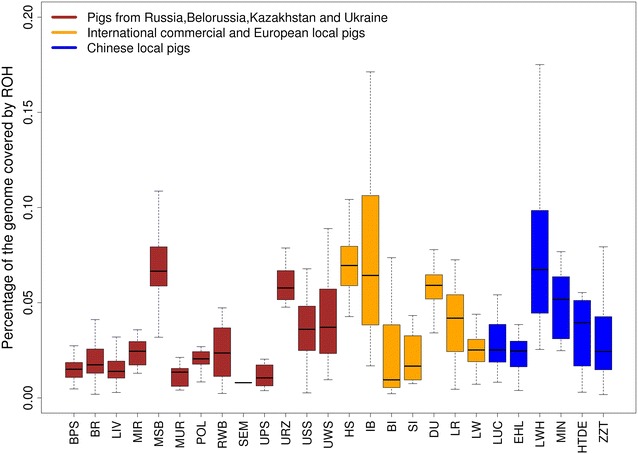
*Box plot* of the distribution of runs of homozygosity for each breed under study.* Each point* denotes the percentage of the genome covered by runs of homozygosity for each individual

The level of ROH reflects the inbreeding history of a population [15]. The Minisib breed had the highest level of ROH (173.3 Mb), followed by the Urzhum (150.3 Mb), Ukrainian White Steppe (93.9 Mb), and Ukrainian Spotted Steppe breeds (93.7 Mb) (Table 1; Fig. 3). In contrast, the other breeds generally had lower levels of ROH than the international commercial and Chinese breeds. The breeds that had the lower levels of ROH and thus lower levels of inbreeding, included the Semirechensk (13.4 Mb), Ukrainian pork swine (27.3 Mb), Murom (32.4 Mb), Livni (36.1 Mb), and Belorussian pork swine (39.1 Mb) breeds (Table 1). For all these breeds, an admixed composition of ancestries at K = 19 was observed in the admixture analysis, which indicates that their increased genetic diversity and reduced levels of ROH can be attributed to their complex crossbreeding history that involved multiple founder breeds.

The average effective population size (Ne) for the past 20 years was estimated for each population using the linkage disequilibrium and recombination rate data. The breeds with the largest Ne included the Breitov (148.7), Belorussian pork swine (145.8), and Red White Belted (142.8) breeds, whereas the Murom (86.2), Minisib (99.5), and Ukrainian pork swine (115.4) breeds had the smallest Ne (Table 1). These estimates are generally smaller than those reported for international commercial breeds such as the Large White (214.4), Duroc (207.2) and Landrace (207.5) breeds, and comparable to estimates for indigenous pig breeds in China and Europe, which ranged from 98.2 for Hetao pigs and 122.4 for Iberian pigs to 165.0 for Erhualian pigs.

## Discussion

Analysis of genetic diversity and population structure based on genomic data has proven useful in revealing the demographic history of pig breeds worldwide [4, 5, 14, 27, 28]. However, to date literature on pig breeds from Russia, Belorussia, Kazakhstan and Ukraine is scarce, although these breeds represent excellent genetic resources for the local economy and good materials to study the genetic basis that underlies their adaptation to local climate, feed, pathogens and human preferences. This study is the first effort to describe the population structure and genetic diversity of pig breeds from Russia, Belorussia, Kazakhstan and Ukraine.

We observed no correspondences between geographical and genetic distances among the 13 breeds studied, which is similar to what was reported for pig breeds from South America [5]. In contrast, pig breeds from China were genetically clustered according to their geographical distribution [26]. These results suggest that different breeding schemes were implemented for different breeds, even for breeds that had close geographical origins.

Overall, our analysis shows that most pig breeds from Russia, Belorussia, Kazakhstan and Ukraine are mainly of European origin and harbor different fractions of ancestries from the Large White, Landrace, Duroc and Hampshire breeds. Many of these local breeds, such as the Breitov, Livny, Murom, Ukrainian White Steppe, Urzhum, and Mirgorod breeds, were officially recognized in the mid-twentieth century (see Additional file 1: Table S1) and the start of the crossbreeding events that led to these breeds can be traced back to the beginning of the twentieth century and even to the end of the nineteenth century. For example, Mirgorod pigs are documented to have been created by crossing Berkshire, Middle White, Large White, and Tamworth pigs since 1890 (see Additional file 1: Table S1). Thus, it is reasonable to assume that a large proportion of European ancestries was introduced to Russia before or at the beginning of the twentieth century. It should be noted that, as documented in historical records, the founders of these pig breeds do not include only Large White, Landrace, Duroc and Hampshire animals. Animals from traditional breeds such as the Middle White, Berkshire, Poland China, Tamworth and Mangalitsa breeds from Great Britain, from the Danish Landrace breed, and local wild boars were also used during their development [8]. A more comprehensive sampling of pig breeds, for example from Great Britain, will allow us to resolve the population structure and genetic compositions of pig breeds in Russia, Belorussia, Kazakhstan and Ukraine in more detail.

The results of the admixture analysis largely agreed with the breeding history of the pig breeds under investigation. According to the Academy of Sciences in Novosibirsk, which developed the Minisib breed, it was the result of crossbreeding between Vietnamese indigenous pigs and Large White and Chinese wild boars [13]. However, our results show that the Minisib breed contains ancestries from Large White, Landrace and Luchuan or Tibetan pigs from China. The Luchuan pig is a miniature pig breed that is similar in appearance to indigenous Vietnamese pigs and originated from the Guangxi Province in Southern China, which is adjacent to Vietnam [28]. Thus, Luchuan pigs may share considerable genetic ancestry with the Vietnamese founders of the Minisib breed, which may have inherited its small body size from these Vietnamese founders.

The Ukrainian Spotted Steppe breed shared a large proportion of ancestry with the Ukrainian White Steppe breed (Fig. 2a, K = 19), which supports the documented role of the Ukrainian White Steppe breed in the development of the Ukrainian Spotted Steppe breed. The Mirgorod breed is considered to be one of the founder breeds of the Semirechensk breed, but we found no Mirgorod ancestry in the Semirechensk breed (Fig. 2a, K = 19) [29]. Moreover, the Livni, Murom and Ukrainian Spotted Steppe breeds also shared ancestry with the Mirgorod breed. Overall, these results generally confirm that the initial formation of pig breeds from Russia, Belorussia, Kazakhstan and Ukraine involved the crossbreeding of multiple foreign breeds, including the Large White, Landrace, Duroc, and Hampshire breeds. Some of the earliest developed breeds, such as the Ukrainian White Steppe and Mirgorod breeds, contributed to the generation of new breeds.

In spite of the complex admixture history of most pig breeds, as indicated by the admixture (K = 19) and neighbor-joining tree analyses, many of the 13 pig breeds investigated in this study have retained unique identities and are differentiated from mainstream commercial breeds such as the Large White and Landrace breeds. This can be attributed to several factors, including differences in the origin of the populations, a long period of genetic isolation, and differences in climate and locally available feed between West Russia, Belorussia, Ukraine and west Europe.

We observed widespread Asian ancestries in all 13 pig breeds. Introgression of Asian haplotypes into European breeds, such as the Large White and Landrace breeds, have been reported in several previous studies [1, 30, 31]. The widespread Asian introgression into breeds from Western Russia, Belorussia, and Ukraine probably occurred via transmission from the Large White, Landrace and Duroc breeds since, the amount of Asian ancestry in the 12 breeds (excluding the Minisib breed) was comparable to that in the Large White, Landrace and Duroc breeds. However, we cannot exclude the possibility that Asian pigs were directly involved in founding or posterior crossbreeding events with the local breeds from Russia, Belorussia and Ukraine.

A previous comparison of genomic diversity parameters based on whole-genome sequence (WGS) and 60K SNP data showed that while 60K SNP and WGS data provided similar results for European breeds, 60K SNP data underestimated the genetic diversity of the Asian populations due to ascertainment bias [32]. This study also demonstrated that results from ROH analyses based on 60K SNP data were generally consistent with those obtained from WGS data [32]. Thus, the levels of ROH that we estimated here should reflect the inbreeding history of the breeds investigated. We observed high levels of ROH and low levels of genetic diversity in the Minisib and Urzhum breeds, which indicates that strong population bottlenecks or inbreeding may have occurred in these breeds. The Minisib breed was subjected to intensive selection for small body size and the Urzhum breed is an indigenous Russian breed, which was not widely intercrossed with other breeds for conservation purposes; these actions may have increased the extent of inbreeding within these populations. Since excessive inbreeding can reduce the long-term fitness of a population, special preservation programs should be implemented to avoid further inbreeding in these breeds. In contrast, the genetic diversity estimated for the other studied breeds was greater than or comparable to that of Chinese pig breeds, which can be attributed to the fact that multiple founder breeds were used to create these breeds. Moreover, since most of these breeds were formed in the mid-twentieth century [9], the sweeping of genetic diversity by selection or genetic drift could have been limited by their short breeding history.

## Conclusions

This is the first genetic survey of pig breeds from Russia, Belorussia, Kazakhstan and Ukraine using 60K SNP genotype data. We confirmed that these breeds were mainly of European origin, since they formed a separate group from Chinese breeds but were close to commercial breeds, including the Large White, Landrace, Duroc and Hampshire breeds. The Minisib and Urzhum breeds have been subject to severe inbreeding and consequently have limited genetic diversity. In contrast, most of the other breeds have a greater level of genetic diversity and a lower level of ROH because of their crossbreeding histories. This study provides the first genomic survey of the population structure and evolutionary history of pig breeds in Russia, Belorussia, Kazakhstan and Ukraine, which will contribute to conservation and breeding programs of these breeds.

## Additional files

10.1186/s12711-016-0196-y Breeding history, average productivity, exterior parameters and characteristics of 13 breeds from Russia, Belorussia, Kazakhstan and Ukraine. The table provides detailed information, including year of official registration, breeding history, average body height and weight, and other characteristics on the 13 breeds under study.
10.1186/s12711-016-0196-y Cross-validation errors of admixture analysis at different K values. This figure displays the distribution of cross-validation errors at different K values in the admixture analysis.

## References

[1] Giuffra E, Kijas JMH, Amarger V, Carlborg O, Jeon JT, Andersson L (2000). The origin of the domestic pig: independent domestication and subsequent introgression. Genetics.

[2] Kijas JMH, Andersson L (2001). A phylogenetic study of the origin of the domestic pig estimated from the near-complete mtDNA genome. J Mol Evol.

[3] Larson G, Dobney K, Albarella U, Fang M, Matisoo-Smith E, Robins J (2005). Worldwide phylogeography of wild boar reveals multiple centers of pig domestication. Science.

[4] Ai H, Huang L, Ren J (2013). Genetic diversity, linkage disequilibrium and selection signatures in chinese and Western pigs revealed by genome-wide SNP markers. PLoS One.

[5] Burgos-Paz W, Souza CA, Megens HJ, Ramayo-Caldas Y, Melo M, Lemus-Flores C (2013). Porcine colonization of the Americas: a 60 k SNP story. Heredity (Edinb).

[6] Laval G, Iannuccelli N, Legault C, Milan D, Groenen MA, Giuffra E (2000). Genetic diversity of eleven European pig breeds. Genet Sel Evol.

[7] Yang SL, Wang ZG, Liu B, Zhang GX, Zhao SH, Yu M (2003). Genetic variation and relationships of eighteen Chinese indigenous pig breeds. Genet Sel Evol.

[8] Shishkov VP (2002). Pig breeding.

[9] Koziner AB, Shtakelberg ER (1989). Animal genetic resources of the USSR.

[10] Dobrokhotov GN (1974). Pig breeding.

[11] Ladan PE, Mysik AT (1981). Pig breeds.

[12] Pocherniaev FK (1990). Breeding, selection and reproduction of pigs.

[13] Nikitin SV, Knyazev SP, Shatokhin KS (2014). Miniature pigs of ICG as a model object for morphogenetic research. Russ J Genet Appl Res.

[14] Herrero-Medrano JM, Megens HJ, Groenen MA, Ramis G, Bosse M, Perez-Enciso M (2013). Conservation genomic analysis of domestic and wild pig populations from the Iberian Peninsula. BMC Genet.

[15] Bosse M, Megens HJ, Madsen O, Crooijmans RP, Ryder OA, Austerlitz F (2015). Using genome-wide measures of coancestry to maintain diversity and fitness in endangered and domestic pig populations. Genome Res.

[16] Ramos AM, Crooijmans RP, Affara NA, Amaral AJ, Archibald AL, Beever JE (2009). Design of a high density SNP genotyping assay in the pig using SNPs identified and characterized by next generation sequencing technology. PLoS One.

[17] Purcell S, Neale B, Todd-Brown K, Thomas L, Ferreira MA, Bender D (2007). PLINK: a tool set for whole-genome association and population-based linkage analyses. Am J Hum Genet.

[18] Weir BS, Cockerham CC (1984). Estimating F-statistics for the analysis of population structure. Evolution.

[19] Yang J, Lee SH, Goddard ME, Visscher PM (2011). GCTA: a tool for genome-wide complex trait analysis. Am J Hum Genet.

[20] PHYLIP: phylogeny inference package. Release: 3.695 (2015). http://evolution.genetics.washington.edu/phylip.html. Accessed 5 Nov 2015.

[21] FigTree: tree figure drawing tool. Release: 1.4.2 (2014). http://tree.bio.ed.ac.uk/software/figtree/. Accessed 12 Nov 2015.

[22] Alexander DH, Novembre J, Lange K (2009). Fast model-based estimation of ancestry in unrelated individuals. Genome Res.

[23] Szpiech ZA, Jakobsson M, Rosenberg NA (2008). ADZE: a rarefaction approach for counting alleles private to combinations of populations. Bioinformatics.

[24] Sved JA (1971). Linkage disequilibrium and homozygosity of chromosome segments in finite populations. Theor Popul Biol.

[25] Tortereau F, Servin B, Frantz L, Megens HJ, Milan D, Rohrer G (2012). A high density recombination map of the pig reveals a correlation between sex-specific recombination and GC content. BMC Genomics.

[26] Megens HJ, Crooijmans RP, San Cristobal M, Hui X, Li N, Groenen MA (2008). Biodiversity of pig breeds from China and Europe estimated from pooled DNA samples: differences in microsatellite variation between two areas of domestication. Genet Sel Evol.

[27] Wilkinson S, Lu ZH, Megens HJ, Archibald AL, Haley C, Jackson IJ (2013). Signatures of diversifying selection in European pig breeds. PLoS Genet.

[28] Pham LD, Do DN, Nam LQ, Van Ba N, Minh LT, Hoan TX (2014). Molecular genetic diversity and genetic structure of Vietnamese indigenous pig populations. J Anim Breed Genet.

[29] Savich EA (2001). Pig breeding.

[30] Bosse M, Megens HJ, Frantz LA, Madsen O, Larson G, Paudel Y (2014). Genomic analysis reveals selection for Asian genes in European pigs following human-mediated introgression. Nat Commun.

[31] Ai H, Fang X, Yang B, Huang Z, Chen H, Mao L (2015). Adaptation and possible ancient interspecies introgression in pigs identified by whole-genome sequencing. Nat Genet.

[32] Herrero-Medrano JM, Megens HJ, Groenen MA, Bosse M, Perez-Enciso M, Crooijmans RP (2014). Whole-genome sequence analysis reveals differences in population management and selection of European low-input pig breeds. BMC Genomics.

