# Erratum to: Population structure and genome characterization of local pig breeds in Russia, Belorussia, Kazakhstan and Ukraine

**DOI:** 10.1186/s12711-016-0235-8

**Published:** 2016-08-11

**Authors:** Aleksei Traspov, Wenjiang Deng, Olga Kostyunina, Jiuxiu Ji, Kirill Shatokhin, Sergey Lugovoy, Natalia Zinovieva, Bin Yang, Lusheng Huang

**Affiliations:** 1 L.K. Ernst Institute for Animal Husbandry, Dubrovitzy 60, Podolsk district, Moscow region Russia 142132; 2 National Key Laboratory for Swine Genetics, Breeding and Production Technology, Jiangxi Agricultural University, Nanchang, 330045 People’s Republic of China; 3 Siberian Research Institute for Animal Husbandry, Russian Agricultural Academy, Novosibirsk, Russia 63050; 4 Mykolayiv National Agrarian University, 9, Paryzka Komuna Str., Mykolayiv, 54020 Ukraine

## Erratum to: Genet Sel Evol (2016) 48:16 DOI 10.1186/s12711-016-0196-y

After publication of this work [1], we noticed that there were some errors in the description of the ROH values on page 6. The values of ROH described on page 6 do not agree with those listed in Table 1. We checked the data and found that the ROH values described on page 6 were from our initial results before the first revision of the manuscript. After the first revision, we added and removed some breeds, redid the quality control procedures and analyses, and obtained the results in Table 1 which differ slightly from the initial results.

The correct description of the ROH values is provided below:

The level of ROH reflects the inbreeding history of a population [15]. The Minisib breed had the highest level of ROH (181 Mb), followed by the Urzhum (148 Mb), Ukrainian White Steppe (104 Mb), and Ukrainian Spotted Steppe breeds (94 Mb; Table 1; Fig. 3). In contrast, the other breeds generally had lower levels of ROH than the international commercial and Chinese breeds. The breeds that had the lower levels of ROH and thus lower levels of inbreeding included the Semirechensk (21 Mb), Murom (30 Mb), Ukrainian pork swine (31 Mb), Livni (39 Mb), and Belorussian pork swine (39 Mb) breeds (Table 1).
