# Supplementary material for: Population structure and genome characterization of local pig breeds in Russia, Belorussia, Kazakhstan and Ukraine
Source: Genet Sel Evol. 2016 Mar 1;48:16. doi: 10.1186/s12711-016-0196-y (PMC4772533; doi:10.1186/s12711-016-0196-y)
Supplement: Supplementary file 1 — 10.1186/s12711-016-0196-y Breeding history, average productivity, exterior parameters and characteristics of 13 breeds from Russia, Belorussia, Kazakhstan and Ukraine. The table provides detailed information, including year of official registration, breeding history, average body height and weight, and other characteristics on the 13 breeds under study. [file 12711_2016_196_MOESM1_ESM.docx]

**Additional file 1:** Table S1. Breeding history, average productivity, exterior parameters and characteristics of 13 pig breeds from Russia, Belorussia, Kazakhstan and Ukraine

| Breed | Year of Official Registration | Breeding history | Body length (cm)^1^ | Body weight (kg) | Attributes |
| --- | --- | --- | --- | --- | --- |
| Belorussian Pork Swine | 1975 | from the end of 19^th^ century, it was created by crossing local Large-white pigs with Tamworth and Landrace | 180/162 | 315/245 | High meat yield, strong body conformation, resistance to diseases, well-adapted for industrial technology of breeding, good meat quality |
| Breitov | 1948 | Prior to the 1917 revolution, cross breeding using Large-white Middle-white, Lithuania-white and  Danish Landrace | 177/161 | 297/236 | Medium-sized body, wide head with a dished face, large drooping ears, wide and deep chest, wide back and loin, hard and sometimes wrinkled skin and dense bristles. The coat-colour is white |
| Poltava | 1993 | Cross breeding started at 1966, using Large-white, Mirgorod, Pietrain, Landrace and Wessex-Saddleback as founders | 186/169 | 330/240 | Wide and long body with well-developed forms of meat. Back is straight. Ham is massive. The coat-colour is white. Poltava pigs are well adapted to forage and high temperature of habit |
| Livny | 1949 | Crossing of the flipped ear Large-white and Landrace started at 1930 | 179/163 | 295/237 | Short and wide head with dished face. Large, thick and slightly drooping ears; wide, straight and sometimes arched back; wide and deep chest; rough skin sometimes with wrinkles; hairy body. The coat-colour is white or black pied; some animals are red-pied or black |
| Minisibs | 1985 | First try of cross breeding started at 1974, by crossing Asian wild boar,Vietnamese indigenous pigs and Large-white | 86/79 | 70/50 | Small body-size, low weight, body conformation close to wild boar, fat rate is low. Used like laboratory model object for research in human diseases. Сan be used as donor of heart valves |
| Murom | 1957 | Crossbreeding started at 1950 in the Central Russia using Lithuania-white and Large-white | 182/167 | 314/257 | Small and light head with slightly dished face, moderately large forward drooped ears, wide and deep chest. Dense bristles growing evenly all over the body. The coat-colour is white |
| Semirechensk | 1978 | First try of crossbreeding started at 1968 in Kazakstan by crossing Large-white,Wild-boar and Kemerovo-breed | 169/155 | 275/222 | Straight face, small erect ears, moderately long body, deep chest, straight and wide back and strong legs. The coat-colour is white; however red, dark brown and black-pied offspring may sometimes occur |
| Ukrainian Pork Swine | 1993 | Crossbreeding started at 1978 using breeds included Large-white, Mirgorod, Landrace, Wessex-Saddleback, Pietrain, and Ukrainian White Steppe | 184/169 | 320/242 | High-yield of meat in the body , thin fat layer; long, wide and deep trunk, well-developed and strong constitution hams. Chest is flat. Coat-colour is white |
| Ukrainian Spotted Steppe | 1961 | Started from 1954, cross breeding using Askaina-Nova bred Ukrainian White Steppe, Berkshire and Mangalitsa pigs | 183/164 | 322/237 | Large body, thick fat type of body conformation with deep chest, straight and wide back. Hams are well developed and rounded. Colour is spotted black-and-white or black-and-tan and sometimes black |
| Ukrainian White Steppe | 1934 | Breeding of first pork pig-breeds of pigs in Ukraine was carried out between 1926-1934 by crossing Large-white, local Large-white flipped-ear pigs and Wild-boars | 183/164 | 332/239 | Medium-sized head with slightly dished face, large ears slightly drooping over the eyes, denser bone, deeper and wider body, strong legs, compact skin and dense bristles. The coat-colour is white |
| Urzhum | 1957 | Crossing Large-white Landrace and Wild-boar from 1948 | 179/163 | 291/245 | Big head with long snout; heavy ears slightly tilted forward; long, deep but not wide body; strong legs; massive and coarse bone; dense bristles. Coat-colour is white |
| Mirgorod | 1940 | From 1890, crossing Berkshire,  Middle-white, Large-white and  Tamworth pigs | 170/153 | 275/217 | Medium-sized head with slightly dished face; small erect ears wide chest; straight wide back; strong medium-long legs which are shorter than in the Large White; hard elastic unwrinkled skin; dense bristles growing evenly all over the body. The coat-colour is predominantly black-pied, but black, black-and-tan, and tan animals sometimes occur |
| Red-White Belted | 2006 | A new pork pig-breed in Ukraine, crossbreeding started from 1994. with Large-white, Landrace, Mirgorod, Wessex-Saddleback, Pietrain, Duroc, and Hempshire | 184/168 | 315/245 | Large body, well-developed meat forms, light head, small ears, horizontal, long, broad and deep body on sturdy limbs. The animals suit of red with a narrow white belt at the shoulder blade area |

^1^ male/female
