# Supplementary figures and images for: Population structure and genome characterization of local pig breeds in Russia, Belorussia, Kazakhstan and Ukraine
Source: Genet Sel Evol. 2016 Mar 1;48:16. doi: 10.1186/s12711-016-0196-y (PMC4772533; doi:10.1186/s12711-016-0196-y)

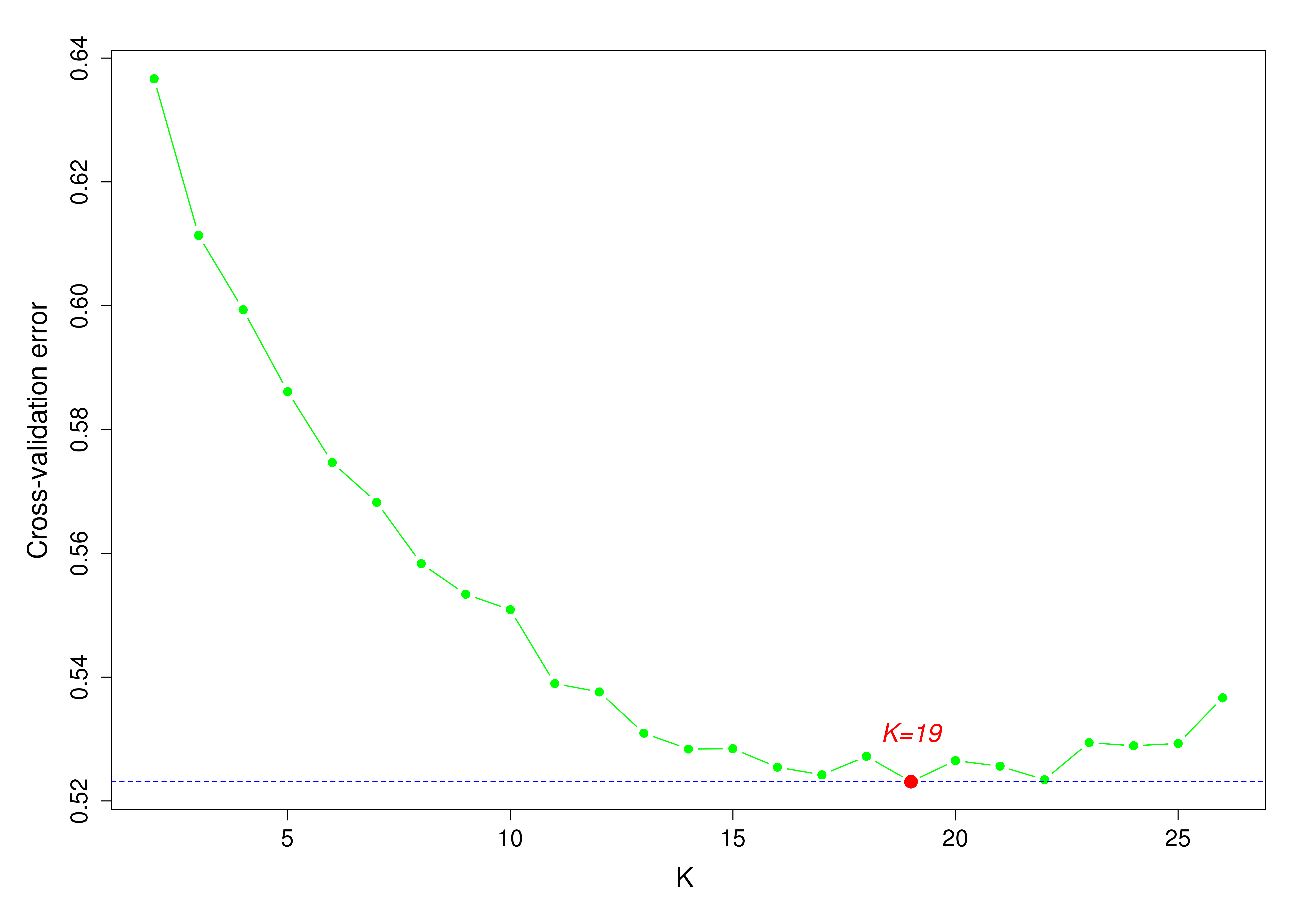

Supplement: Supplementary file 2 — 10.1186/s12711-016-0196-y Cross-validation errors of admixture analysis at different K values. This figure displays the distribution of cross-validation errors at different K values in the admixture analysis. [file 12711_2016_196_MOESM2_ESM.tif]
